# New Insights into the Cellular Toxicity of Carbon Quantum Dots to *Escherichia coli*

**DOI:** 10.3390/antiox11122475

**Published:** 2022-12-16

**Authors:** Shirong Qiang, Li Zhang, Zhengbin Li, Jianjun Liang, Ping Li, Jiayu Song, Kunling Guo, Zihuan Wang, Qiaohui Fan

**Affiliations:** 1 Key Laboratory of Preclinical Study for New Drugs of Gansu Province, Institute of Physiology, School of Basic Medical Sciences, Lanzhou University, Lanzhou 730000, China; 2 Second Clinical Medical College of Lanzhou University, Lanzhou University, Lanzhou 730000, China; 3 Northwest Institute of Eco-Environment and Resources, Chinese Academy of Sciences, Lanzhou 730000, China; 4 School of Public Health of Lanzhou University, Lanzhou University, Lanzhou 730000, China

**Keywords:** carbon quantum dots, *E. coli*, biologic toxicity, membrane permeability, osmotic pressure

## Abstract

In this study, the cytotoxicity and toxic mechanism of carbon quantum dots (CQDs) to *E. coli* were evaluated in vitro. The synthetic CQDs were extremely small in size (~2.08 nm) and displayed strong fluorescence. The results demonstrated that CQDs showed good biocompatibility with *E. coli* within a short culture time. However, when the exposure time exceeded 24 h, the toxicity of CQDs became apparent, and the contents of reactive oxygen species, lactate dehydrogenase, and the crystal violet absorption rate increased significantly. To further explore the cytotoxic mechanism, approaches including confocal laser scanning microscopy, scanning electron microscopy, and biological transmission electron microscopy combined with zeta potential tests, osmotic pressure measurement, and comet assays were performed. On the one hand, the CQDs altered the surface charges of cells and induced lipid peroxidation by adhesion on the surface of *E. coli*, leading to an increase in the permeability of the cell wall. On the other hand, when the concentration of CQDs reached 200 µg/mL, the osmotic pressure of the extracellular environment was significantly reduced. These are the main factors that lead to cell edema and death. Finally, the comet assays confirmed that CQDs could induce DNA damage, which could inhibit the proliferation of *E. coli*.

## 1. Introduction

Owing to their potential in a variety of application technologies, carbon nanomaterials such as fullerenes, carbon nanotubes, graphene sheets, and carbon quantum dots (CQDs) have inspired extensive research [1]. CQDs can be synthesized and functionalized quickly and easily [2] and are recognized for their low cost and chemically inert nature [1,2,3]. CQDs mainly refer to surface-modified or functionalized fluorescent or luminescent carbon nanoparticles with a particle size of less than 10 nm [2]. These are typical quasi-spherical nanoparticles, generally composed of graphite or turbo-layered carbon (sp^2^ carbon) or diamond-like sp^3^ hybrid carbon fused by graphene and graphene oxide flakes [1]. Owing to its small particle size, strong tunable fluorescence characteristics, high photoluminescence emission, and the properties of oxygen functional groups, CQDs have broad application prospects in photocatalysis, ion sensing, biological imaging, heavy metal detection, adsorption treatment, film preparation, and water pollution treatment. Therefore, CQDs will be inevitably released into the environment and may have toxicological effects on the ecosystem and human beings.

Previous studies have reported the inhibitory effect of CQDs and N-doped CQDs on the growth of the microalga *Chlorella pyrenoidosa* and confirmed the toxicities of CQDs and N-doped CQDs even at the low concentration of 50 mg/L [4]. Using Hepal-6 cells as a model, Zhang et al. demonstrated that N-doped CQDs were located in lysosomes and induced reactive oxygen species (ROS)-mediated cellular protective autophagy at high doses (>100 µg/mL) [5]. Moreover, Yan et al. found that the toxicity of CQDs was related to their charge density, where the more positive charges on CQDs, the greater the toxicity to human umbilical cord-derived mesenchymal stem cells [6]. Zhang et al. reported that CQDs may affect the water content of a fungus ball [7]. Xiao et al. found that CQDs exposure can reduce Na^+^-K^+^-ATPase activity in a concentration-dependent manner that could prevent Na^+^-K^+^ pumping and thereby disrupt the balance of Na^+^ accumulation, eventually leading to cell edema and destruction [8]. In addition, the decrease of CQDs size could lead to a huge increase in the surface-to-volume ratio, thereby allowing a greater number of molecules to adhere to the surface of the cell and enhancing its intrinsic toxicity [9]. Navarro-Ruiz et al. have evaluated the characteristics and nanotoxicity of CQDs in fibroblasts and hepatocytes, and found different pathways for the interaction of CQDs with these two types of cells [10]. Significantly, in terms of their internalization capacity and toxicity, the responses in cell type-specific and micro-environmental clues are two key factors [10,11]. Therefore, the cytotoxicity and mechanism of CQDs should be quite different for various cell types. However, the toxicity process and mechanism of CQDs to various cells still remain unclear.

CQDs are considered to be highly biocompatible materials with fluorescence properties, which will be, as expected, widely used in the medical domain, for example, tumor localization and in vivo drug delivery. *Escherichia coli* (*E. coli*) is a resident flora in the human body, and it will lead to the imbalance of intestinal flora and threaten intestinal health when the number is unbalanced. Therefore, a detailed study of the toxic mechanism of CQDs against *E. coli* to determine safe use concentrations is essential for the future safe applications of CQDs. In addition, *E. coli* is a gram-negative bacterium that has been widely used as a model organism for nanotoxicology studies. Previous studies have shown that CQDs have a certain toxic effect on the growth of *E. coli* even at low concentrations, but the study on bacterial damage is still deficiency, especially at a microscale [12]. Moreover, the toxicity of CQDs may be related to the surface charge of the material and the induced production of reactive oxygen species [13]. However, the specific toxicity mechanism of CQDs to *E. coli* and the reasons for the changes in toxicity level are still unclear. In this study, *E. coli* was chosen as the biological model. The toxicity of CQDs to *E. coli* was studied and analyzed, and the findings will provide a theoretical basis for a wider range of safe applications of CQDs.

## 2. Materials and Methods

### 2.1. Strains and Reagents

In this study, the *E. coli* ATCC25922 strain was obtained from the Lanzhou Institute of Animal Husbandry and Veterinary Medicine, Chinese Academy of Agricultural Sciences. CQDs were prepared through a simple bottom-up method [14]. The relevant media and chemicals were analytical grade and untreated before use.

### 2.2. Characterization of CQDs

The morphology and size of CQDs were characterized using a high-resolution transmission electron microscope (TEM, Tecnai F30) under the accelerating voltage of 300 kV. The samples were prepared by the deposition of 10 μL of CQDs aqueous solution on a copper grid coated with an ultra-thin film of carbon, while the solvent was removed by evaporation in the air at room temperature. The fluorescence images of CQDs were obtained under the irradiation of UV light with a wavelength of 254 nm. The fluorescence spectra of CQDs in water were collected under different excitation wavelengths using a fluorescence spectrometer (FLS920, Edinburgh Instruments).

### 2.3. Bacteriostatic Experiment

*E. coli* solutions incubated for 24 h were adjusted to OD_600_ = 0.1 using LB liquid medium. The OD_600_ was measured by placing the bacterial suspension in a 96-well plate using a microplate reader (BIOBASS-EL10B). CQDs were mixed with the above culture solutions to achieve different doses of CQDs (0, 1, 10, 20, 50, 100, and 200 μg/mL), and then cultured in a shaking table at 37 °C and 220 rpm/min. The OD_600_ value of the mixed culture medium was measured every 2 h and used to construct a growth curve.

The above samples were cultured for 2 h and diluted in multiples of 1:100,000, and then 100 µL of the diluted sample was taken in solid medium plates and cultured for 18 h at 37 °C. Finally, the number of colony-forming units in the plates were counted.

### 2.4. MTT Assay

The survival rate of *E. coli* after exposure to CQDs was measured by the MTT method. Diluted *E. coli* were cultured overnight until the OD_600_ was equal to 0.1, and then mixed with different doses of CQDs (0, 20, 50, 100, and 200 μg/mL). After being cultured for different times (24, 48, and 72 h) in a shaking table at 37 °C and 220 rpm/min, 200 µL of the above culture was sampled, and then 200 µL MTT and 800 µL PBS were added. After incubation in a water bath at 37 °C for 30 min and centrifugation (10,000 rpm/min) for 5 min, the supernatant was discarded, and 3 mL DMSO was added. Finally, after centrifugation at 4 °C and 10,000 rpm/min for 5 min, 200 µL of the supernatant was taken to measure the absorbance value at a wavelength of 490 nm.

### 2.5. Oxidative Stress Detection

The lipid oxidation level was determined through MDA content. In brief, the mixtures of *E. coli* (OD_600_ = 0.1) and CQDs (0, 20, 50, 100, 200, and 500 μg/mL) cultured at different times were centrifuged at 4 °C and 4000 rpm for 5 min, and then washed three times with PBS solution. After that, 1.0 mL of malondialdehyde extract was added. The solutions were then subjected to ultrasonic decomposition for 10 min (20% power) and centrifugation at 4 °C and 8000 rpm for 10 min to collect the supernatant. Then, 0.1 mL samples were mixed with 0.3 mL 0.5 mol/L HCl and 0.33 mL 0.67% TBA reagent, and heated at 95 °C for 30 min. After the samples were cooled to room temperature, 4.0 mL of methanol was added, and the solution was shaken for 45 min. Finally, the absorbance of the collected upper liquid was measured at 535 nm.

The oxidative stress induced by CQDs could be determined by the content of hydrogen peroxide produced under the exposure to different doses of CQDs (0, 20, 50, 100, 200, and 500 μg/mL). Briefly, CQDs and *E. coli* were mixed and incubated for a fixed time; after being washed with PBS two times, the cell lysate was collected, and then the reaction solution (phenolic red 0.56 mmol/L, horseradish peroxidase 17 µmol/mL) was introduced. After 2 min, 10 µL 0.5 M NaOH was added to stop the above reaction, and then the absorbance at 630 nm was measured.

### 2.6. Interaction of CQDs with the Bacterial Membrane

By measuring the content of LDH in the final samples, we demonstrated that CQDs could interact with the *E. coli* membrane and destroy it. After *E. coli* (OD_600_ = 0.1) was exposed to CQDs (0, 20, 50, 100, 200, and 500 μg/mL), 10 mL of mixed culture medium was taken for LDH measurement.

The damage to the bacterial membrane was evaluated by the crystal violet uptake method. The collected samples with different doses of CQDs (0, 20, 50, 100, 200, and 500 μg/mL) and *E. coli* were centrifuged at 9300× *g* for 5 min and re-suspended in 0.5% NaCl solution containing 10 µg/mL crystal violet. After incubation at 37 °C for 10 min, the sample was centrifuged at 13,400× *g* for 15 min, and the supernatant was taken to measure the OD_590_ value by a UV-vis spectrophotometer. The above measurements were made in triplicate, and the crystal violet content was calculated by the following formula: crystal violet absorption = (sample OD_590_ value)/(crystal violet solution OD_590_ value) × 100%.

A confocal laser scanning microscope (CLSM) (Nikon A1R+Ti2-E) was used to observe the interaction between CQDs and the *E. coli* membrane. *E. coli* (OD_600_ = 0.1) were exposed to 500 μg/mL CQDs for 72 h. After being stained with 1.0 mL of 10 μmol/mL Dil dye and washed three times by PBS, the cells were dropped onto microscope slides, and observed with CLSM under light-protected conditions at excitation wavelengths of 549 nm and 380 nm, respectively.

### 2.7. SEM and TEM Characterizations

The morphological changes of *E. coli* after exposure to CQDs (0, 100, and 500 μg/mL) were observed by scanning electron microscopy (SEM) and biological transmission electron microscope (TEM). Freeze-dried SEM samples were placed in conductive adhesive and coated with gold for SEM observation.

For biological TEM measurement, *E. coli* was sliced into ultrathin sections. In brief, *E. coli* was firstly cultured overnight with different doses of CQDs (0, 50, 100, 200, 300, and 500 μg/mL), and then incubated at 37 °C and 220 rpm for 72 h. After being centrifugated at 8000× *g* for 5 min, the supernatant was discarded, and the solid samples were fixed with 2.5% glutaraldehyde for 2 h, then washed with a PBS buffer, and finally dehydrated with a gradient series of ethanol with various concentrations. The obtained samples were then dehydrated twice with anhydrous ethanol and acetone, and then embedded into resin overnight. The samples and embedding agent were placed into a mold, and then polymerized in an oven at 35 °C for 12 h, 45 °C for 12 h, and 60 °C for 48 h. Subsequently, an ultrathin microtome was used to slice the samples to a thickness of 50–70 nm, and then the slices were observed under a biological TEM after double staining with lead and uranium.

### 2.8. Comet Assay

The DNA damage to *E. coli* caused by CQDs was evaluated by single-cell gel electrophoresis (known as the comet assay). *E. coli* were placed in the culture medium containing 500 μg/mL CQDs for 72 h and centrifuged at 8000 rpm for 10 min, and then washed twice with PBS buffer. After adding the same amount of PBS buffer, nine times the volume of 0.75% low-melting agarose was added at 37 °C, and the sample was allowed to stand for 20 min at 4 °C. After drying, the samples were put into a lysis solution for 1.5 h, washed twice with deionized water, and then put into SUB-CELL GT. The slides were placed in an electrophoresis tank, and the TBE buffer (Tris, borate, and EDTA) was added to the electrophoresis for 30 min at 30 V. After electrophoresis, the samples were neutralized in 0.4 M tris-HCl for 5 min, and then dehydrated in anhydrous ethanol. The prepared samples were stained with an EB dye solution of 20 mg/L for 5 min, then washed with deionized water. Finally, the samples were observed under a fluorescence microscope at the excitation wavelength of 535 nm.

### 2.9. Zeta Potential and Osmotic Pressure Measurements

In brief, *E. coli* was exposed to a CQD solution (0, 20, 50, 100, 200, and 500 μg/mL) for 72 h where the pH was controlled at 7.0 using a dilute HNO_3_ or NaOH solution, and then the zeta potential was immediately measured using a zeta potential analyzer (Zetasizer Nano ZS analyzer, Malvern, UK). After *E. coli* was exposed to a CQD solution (0, 50, 100, 200, 300, and 500 μg/mL) for 72 h, the osmotic pressure was determined using an osmometer (6003 Touch Micro Osmette, Precision Systems Inc., Tech Circle, Natick, MA, 01760, USA).

## 3. Results

### 3.1. Characterization of CQDs

**Figure 1:**
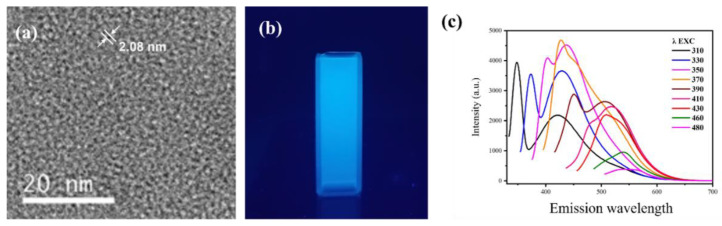
Characterizations of carbon quantum dots. (**a**) TEM, (**b**) fluorescence of CQDs under ultraviolet irradiation, and (**c**) fluorescence response of CQDs under different excitation wavelengths.

As shown in Figure 1a, the synthesized CQDs were uniformly dispersed in water and the particle size of CQDs was ~2.08 nm. Under the excitation of a UV light (with a wavelength of 254 nm), the CQDs emitted blue fluorescent light (Figure 1b). As shown in Figure 1c, the emission spectra of CQDs exhibited a typical excitation wavelength-dependent, in which emission peaks red-shifted steadily with the increase in the excitation wavelength.

### 3.2. Bacteriostatic Experiment

**Figure 2:**
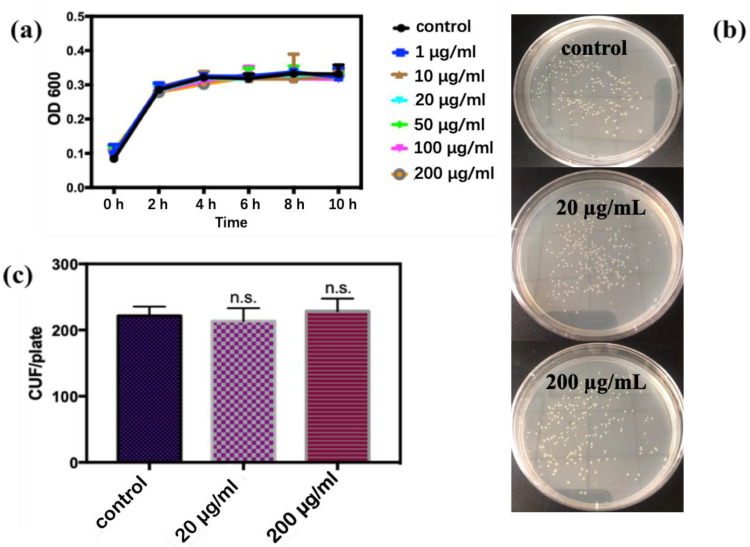
(**a**) Growth curve of *E. coli* in different concentrations of CQDs; (**b**,**c**) bacteriostatic test results and CFU count of exposure to different concentrations of CQDs (n.s. *p* > 0.05).

In this study, the growth curve of *E. coli* was obtained under different concentrations of CQDs and culture periods. As shown in Figure 2a, with the increase in exposure time, the growth rate of *E. coli* was similar to that of the control group within 10 h. The results of bacteriostatic experiments are shown in Figure 2b,c, and there was no significant difference in colony count between the control group and the groups exposed to CQDs. The results suggest that CQDs had no adverse effects to *E. coli* within a short duration of exposure.

### 3.3. MTT Assay

**Figure 3:**
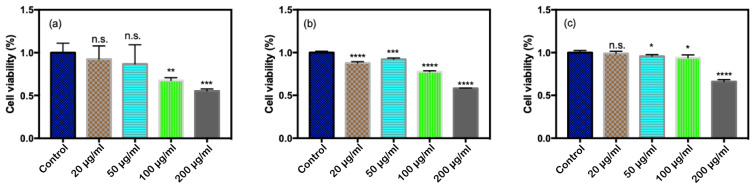
Survival rate of *E. coli* exposed to different concentrations of CQDs measured by MTT method. (**a**) 24 h, (**b**) 48 h, and (**c**) 72 h (n.s. *p* > 0.05, * *p* < 0.05, ** *p* < 0.01, *** *p* < 0.001, and **** *p* < 0.0001).

As shown in Figure 3, after exposure to CQDs for more than 24 h, the survival rate of *E. coli* showed a decreasing trend with the increase in the concentration of CQDs. When the concentration of CQDs reached 200 µg/mL, the survival rate was significantly lower than those of the control group and the low-concentration group. These results showed that with the increase in exposure time, CQDs had a certain lethal effect on *E. coli*, especially at high concentrations of CQDs.

### 3.4. Oxidative Stress

**Figure 4:**
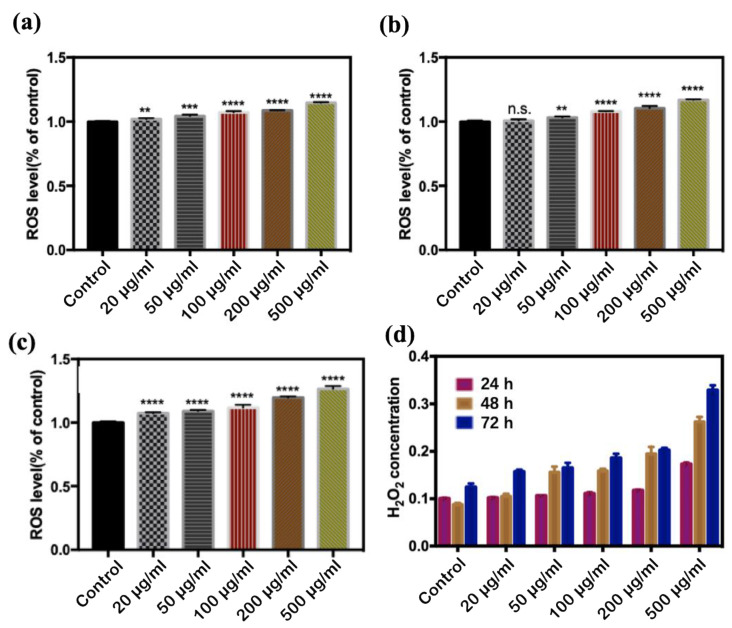
Concentrations of MDA and H_2_O_2_ produced by *E. coli* exposed to different concentrations of CQDs. (**a**) MDA24 h, (**b**) MDA48 h, and (**c**) MDA72 h, (**d**) HRP test (n.s. *p* > 0.05, ** *p* < 0.01, *** *p* < 0.001, and **** *p* < 0.0001).

As can be seen from Figure 4a–c, during the same exposure time, the content of MDA increased gradually with the increase of CQD concentration, indicating that CQDs caused lipid peroxidation in *E. coli.* With the increase of CQD concentration, the content of H_2_O_2_ generated by *E. coli* showed an increasing trend (Figure 4d), which confirms the production of H_2_O_2_ was time- and dose-dependence.

### 3.5. Interaction of CQDs with the Bacterial Membrane

**Figure 5:**
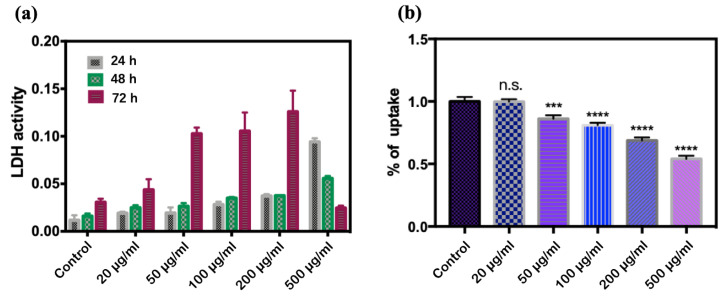
Effect of CQDs on the *E. coli* membrane. (**a**) The amount of LDH released by *E. coli* at different CQDs concentrations; (**b**) the uptake rate of crystal violet (n.s. *p* > 0.05, *** *p* < 0.001 and **** *p* < 0.0001).

From Figure 5a, the activity of LDH increased significantly with the increase in the concentration of CQDs. When the concentration of CQDs was constant, the amount of LDH released by *E. coli* increased with exposure time. However, when the concentration of CQDs was 500 µg/mL, the activity of LDH decreased sharply with the extension of exposure time.

In the absence of CQDs, the mean value of OD_590_ in bacterial suspension was 0.1733, which suggests a high uptake of crystal violet (Figure 5b). With the increase in CQD concentration, the OD_590_ decreased in proportion, and when the exposure concentration of CQDs reached 500 µg/mL, the OD_590_ decreased to 0.0936.

**Figure 6:**
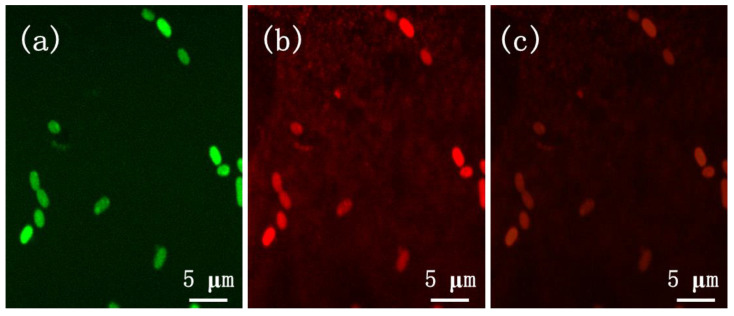
CLSM image of *E. coli*. (**a**) Green fluorescence from CQDs, (**b**) the fluorescence of Dil dye, (**c**) composite image of (**a**,**b**) [CQDs] = 500 μg/mL.

The fluorescence from CQDs was found at the excitation wavelength of 380 nm (Figure 6a), which is consistent with the results of the fluorescence spectrum (Figure 1c). At the same time, the Dil staining on the bacteria could be clearly observed at the excitation wavelength of 549 nm (Figure 6b). One can see from Figure 6a–c, in the CLSM image, the distribution of CQDs was highly correlated to that of bacteria (i.e., *E. coli*), which indicates that the bacteria of *E. coli* has a high affinity to bounding CQDs.

### 3.6. Characterizations of SEM and TEM on E. coli Exposed to CQDs

**Figure 7:**
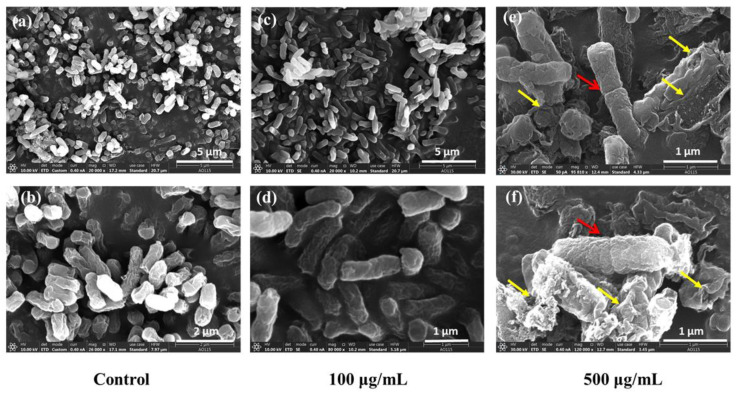
SEM image of *E. coli*. (**a**) and (**b**) Control group, (**c**) and (**d**) 100 μg/mL, (**e**) and (**f**) 500 μg/mL.

In Figure 7, the morphology of bacterial cells in the blank group (Figure 7a,b) and low concentration group (Figure 7c,d) was complete, while some bacteria in the high concentration group (Figure 7e,f) remained in the interphase of division (as indicated by the red arrow in the figure); some cell membranes were broken, and the bacteria were deformed and cracked (as indicated by the yellow arrow).

**Figure 8:**
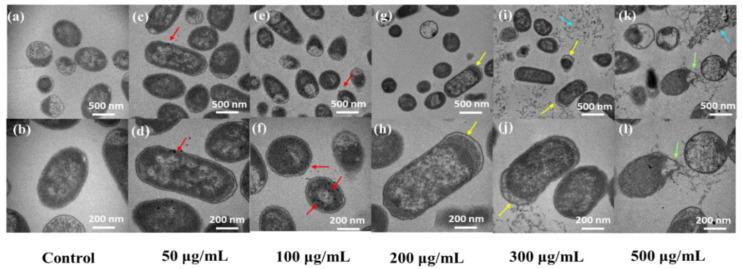
Ultrathin images of *E. coli* using a biological transmission electron microscope. (**a**,**b**) Blank group, (**c**,**d**) 50 μg/mL, (**e**,**f**) 100 μg/mL, (**g**,**h**) 200 μg/mL, (**i**,**j**) 300 μg/mL, and (**k**,**l**) 500 μg/mL.

As shown in Figure 8a,b, the cells in the blank group had normal morphology and a complete internal structure, with the cell wall and cell membrane being closely attached. In Figure 8c–f, some CQDs are shown adhering to the cell membrane and entering the cell interior (as indicated by the red arrow). When the concentration of CQDs reached 200 µg/mL, the cells became swollen and deformed, as indicated by the yellow arrows in Figure 8g,h. With the further increase of CQDs concentration, cell edema became more pronounced (Figure 8i,j), the cell membrane ruptured (as indicated by the green arrows in the figures), and cytoplasm flowed out (as indicated by the blue arrows), accompanied by a large number of dead cells (Figure 8k,l).

### 3.7. Comet Assay

**Figure 9:**
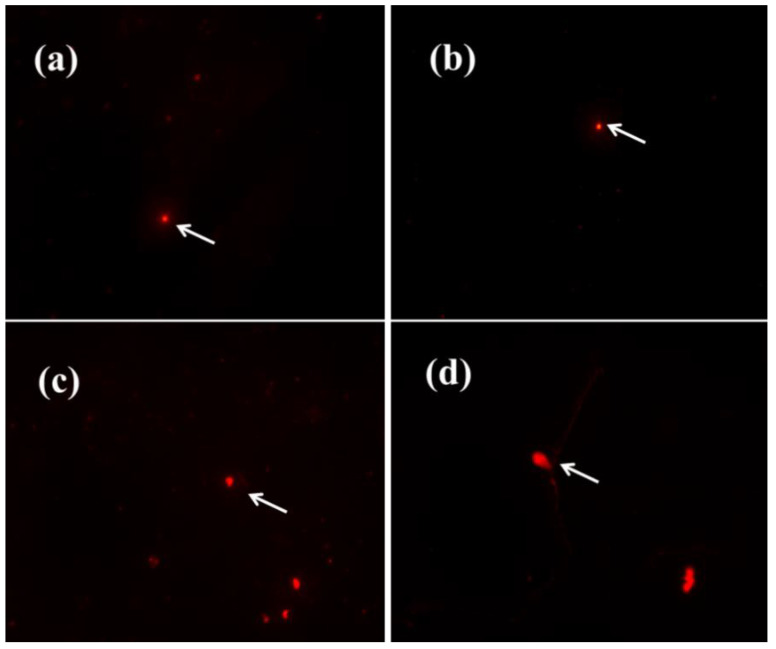
Comet experiment results. (**a**,**b**) Control group, (**c**,**d**) 500 μg/mL.

Compared to the control group (Figure 9a,b), when the CQD concentration was 500 µg/mL, *E. coli* showed a trailing phenomenon (Figure 9c,d), but the comet tail was very small. The results indicate that CQDs could induce DNA fracture in *E. coli*, but the DNA damage was relatively weak.

### 3.8. Zeta Potential and Osmotic Pressure

**Figure 10:**
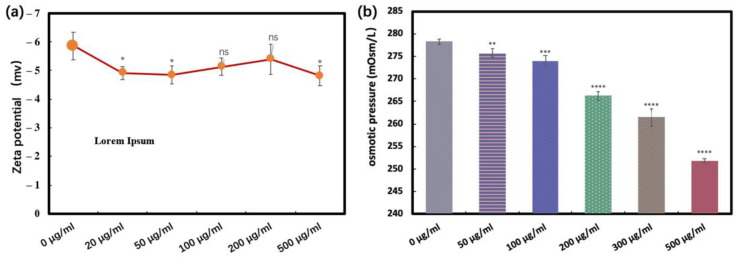
Zeta potential and osmotic pressure of CQD at different concentrations. (**a**) Zeta potential and (**b**) osmotic pressure (n.s. *p* > 0.05, * *p* < 0.05, ** *p* < 0.01, *** *p* < 0.001, and **** *p* < 0.0001).

As shown in Figure 10a, after mixing *E. coli* with CQDs and incubating for a period of time, compared with the blank group, the zeta value of other systems decreased in the negative direction. Figure 10b shows the osmotic pressure formed by different concentrations of CQDs exposed to an LB liquid medium. With the increase in CQDs concentration, the environmental osmotic pressure gradually decreased. With the CQDs concentration of 200 µg/mL as the dividing point, the osmotic pressure decreased significantly and then remained low.

## 4. Discussion

### 4.1. Characterization of CQDs

CQDs are considered to be quasi-spherical carbon nanomaterials with a size of less than 10 nm and capable of stable luminescence [4]. In this study, we used synthetic CQDs with a diameter of about 2.0 nm to explore the toxic effect and mechanisms of CQDs on *E. coli*.

### 4.2. Evaluation of Cytotoxicity of CQDs to E. coli

#### 4.2.1. Analysis of Antibacterial Activity after a Short Exposure Time

Previous studies on the toxicity of carbon nanomaterials, carbon nanotubes, and their derivatives have demonstrated the toxic effects on a variety of organisms, including *E. coli* [15,16]. According to the methods described in previous literature [17,18], we quantified the antibacterial activity of *E. coli* by growth curves and bacteriostatic experiments. As one form of carbon nanomaterials, CQDs have shown biocompatibility in low doses and short exposure times. However, Liu et al. concluded that a high concentration of CQDs would inhibit the growth of *E. coli* to a large extent [12]. Our results clearly suggested that as the dose of CQDs and culture time increased, CQDs still showed obvious toxicity to *E. coli*.

#### 4.2.2. Survival Rate with the Time of Exposure

The cytotoxicity of CQDs was evaluated in vitro by the MTT assay. The reagent 3-(4,5-dimethylthiazole-2)-2,5-diphenyltetrazolium bromide (MTT) is a dye capable of accepting hydrogen atoms. The succinate dehydrogenase in the mitochondria of living cells can restore exogenous MTT to insoluble blue-purple crystals and deposit them in cells, while dead cells cannot. Within a certain range of cell numbers, the amount of MTT crystals is proportional to the number of living cells. Therefore, the survival rate of *E. coli* can be calculated by measuring the amount of generated blue-purple crystals. This indicated that CQDs had a time- and dose-dependent toxic effect on the proliferation of *E. coli*, consistent with previous studies [19,20,21].

#### 4.2.3. Oxidative Stress

Malondialdehyde (MDA) is a lipid peroxidation product produced by oxygen-free radicals and polyunsaturated fatty acids in biofilms. When heated under acidic conditions, it can react with thiobarbituric acid (TBA) to produce a color reaction, and the resulting substance has an absorption peak at 535 nm. In this study, the ability of CQDs to induce oxidative stress in *E. coli* was assessed by measuring the MDA content [22,23]. It had been noted previously that the antimicrobial activity of CQDs resulted from the production of ROS and the release of toxic ions [24]. To demonstrate that CQDs could induce *E. coli* to produce ROS, the phenol red-horseradish peroxidase microplate method was used to measure the amount of H_2_O_2_ produced by *E. coli* after CQD exposure. H_2_O_2_ can oxidize phenol red to form chromophores containing π electrons under the catalysis of horseradish peroxidase. In the presence of exogenous horseradish peroxidase, the color of the phenol red pH indicator in the sample changed from red to yellow, during which a large amount of H_2_O_2_ was produced. When the solution pH was raised to 12.5, the color changed from yellow to purple and remained stable. Our experimental data clearly showed that CQDs could induce ROS in *E. coli* cells and ROS could cause the oxidation of *E. coli* cell membranes, as shown by the determination of MDA as a by-product of lipid peroxidation of unsaturated fatty acids [25]. Therefore, we speculated that long-term exposure to CQDs in high concentrations could lead to somehow damage of cell walls in *E. coli.*

#### 4.2.4. Changes in the Bacterial Cytoderm

To further study the damage to the *E. coli* cell wall induced by CQDs, the LDH and the crystal violet uptake tests were performed. As an enzyme with relatively stable activity, LDH is widely present in the cytoplasm of cells. When the membrane is destroyed and the integrity is lost, LDH will be released from the cytoplasm. Therefore, we evaluated the cytotoxicity of the CQDs by measuring the content of LDH in the supernatant after centrifugation. In the present study, the damage of *E. coli* cell walls by CQDs was dose- and time-dependent. It has been reported that oxidative stress products interact with LDH and inactivate LDH [26]. Therefore, when the CQD concentration reached 500 µg/mL, the LDH content decreased with exposure time, possibly as a result of the reaction between a large amount of ROS produced and LDH. Another possibility is that after exposure for a long time at a high concentration of CQDs, a large amount of *E. coli* will have died, resulting in lower enzyme activity of LDH.

Crystal violet has poor membrane penetration ability, but once the membrane is damaged and the permeability changes, it is easy for it to enter the cell [27,28]. The value of OD_590_ reflected the concentration of crystalline violet in the solution and decreased with the increasing CQDs concentration in this study, which means that crystal violet had infiltrated the *E. coli* and that the permeability of the cell membrane was increased. Although CQDs increased the uptake rate of crystal violet by bacteria, the increase rate was small, indicating that CQDs did not directly damage the cell membrane. This suggested that CQDs could change the permeability of the cell wall by some mechanism. Roy et al. proposed that the high surface potential of nanoparticles can improve their protein binding efficiency, thus promoting the recognition of surface receptors and ultimately achieving internalization [29].

To confirm whether CQDs could lead to cell wall alteration in *E. coli*, we first observed the direct interaction between CQDs and the *E. coli* cytoderm by the Dil staining method and observed the changes under a laser confocal microscope. The results confirmed that CQDs were indeed attached to the membrane surface. Previous studies have shown that carbon nanomaterials acted on the membranes of model organisms through a series of complex mechanisms. For example, Ahmad et al. reported that the sharp edges of CQDs could easily pierce the *E. coli* membrane [30]. Qiang et al. found that graphene oxide ribbons (GORs) could gather around *E. coli* and destroy the cell membranes by extracting components of the cell membrane [31].

### 4.3. Cytotoxic Mechanism of CQDs in E. coli

#### 4.3.1. Microscopic Morphological Analysis

SEM can illustrate the morphological changes of the bacteria. Low concentrations of CQDs have good biocompatibility, while high concentrations of CQDs induce cell deformation and rupture and stunt bacterial multiplication, resulting in the elongation of cell chains and the inability to divide into single cells.

Biological TEM was used to describe the changes in the internal structure of the bacteria. At low concentrations, CQDs have been internalized by the cells without significant cytotoxicity, while high concentrations clearly lead to cellular edema and rupture, cytoplasmic efflux, and cell death, indicating that increasing concentrations lead to toxic accumulation of CQDs. The results indicate that the main reason for the damage and even death of *E. coli* caused by CQDs could be attributed to cell swelling caused by the change in cell permeability. However, the exact mechanism still needs further research. One possible cause of the change in the permeability of *E. coli* is oxidative stress, where the adhesion of CQDs to the bacterial cell wall leads the superoxide anion and free radicals being produced that rapidly and directly interact with bacteria, destroying the cell membrane and causing leakage of the cytoplasm [32,33].

#### 4.3.2. Analysis of DNA Damage

Ghosh et al. [34] reported that multi-walled carbon nanotubes (MWCNTs) could cause chromosome aberration, DNA breakage, and apoptosis in onion root cells, and this may be related to the internalization of MWCNTs in plant cells. Single-cell gel electrophoresis (the comet assay) is a method employed to rapidly detect DNA damage in a single cell. An alkaline electrophoresis solution can cause the double-stranded DNA to unwind and degenerate into a single strand. When single-stranded DNA breaks, the broken fragments can enter the gel due to their small molecular weight and migrate from the nuclear matrix to the anode during electrophoresis, forming a pattern resembling the tail of a comet. Under the same electrophoresis conditions, the longer the tail, the greater the number of broken fragments. When CQDs entered the bacteria, the growth cycle of bacteria was altered; the number of bacteria staying in the splitting plateau increased, and their growth and reproduction were inhibited, although most bacteria remained active. Therefore, together with the SEM results, we conclude that another toxic mechanism of CQDs to *E. coli* is that the CQDs enter *E. coli* and induce a small amount of DNA breakage, thereby inhibiting bacterial reproduction.

#### 4.3.3. Zeta Potential

In gram-negative bacteria, apart from the thin peptidoglycan layer that acts as an additional barrier for the solutes, the presence of a higher density of anionic groups and O-antigen in the LPS membrane aid in the maintenance of the surface potential and in turn support membrane integrity [35]. The change in *E. coli* cell surface potential may cause changes in membrane permeability. Zeta potential has been demonstrated to be a useful method for measuring the surface charge of bacterial cells [36]. Halder et al. [37] found that there was a certain correlation between the alteration of the zeta potential and the change in membrane permeability. When the potential shifts toward neutrality, the bacterial membrane will be destabilized [38]. Changes in the zeta potential of the system may lead to destabilization of the cell wall and changes in permeability.

#### 4.3.4. Changes in Osmotic Pressure of *E. coli*

Li et al. reported that when the concentration of CdTe quantum dots was higher than 70.2 nmol/L, Halo bacillus halogen R1 cells would swell and deform [39]. According to the TEM characterization, we hypothesized that CQDs would change the osmotic pressure of the medium. Changes in the concentration of CQDs resulted in the formation of different osmotic pressures in the LB liquid medium. Cells can detect and balance the changes in extracellular solute concentration; this is called osmoregulation, and it is a basic attribute of all cells [40]. The osmotic stress brought by the change of extracellular osmotic pressure alters the cell volume, resulting in a change in the concentrations of intracellular macromolecules. With the decrease of the extracellular osmotic pressure of *E. coli* and the increase of cell wall permeability, water will enter the cell, causing the cell to swell. As shown in Figure 8g,h, this change occurred when the CQD concentration reached 200 µg/mL, and as the concentration of CQDs increased, this change became more pronounced, consistent with the change in osmotic pressure. Thus, it can be speculated that when the concentration of CQDs is greater than 200 µg/mL, the sharp drop in osmotic pressure would be the main reason for the mass death of cells.

## 5. Conclusions

CQDs have the advantages of easy synthesis, strong fluorescence, high solubility and dispersion, and biosafety, indicating their research value in the medical field. For example, new orange-emissive carbon quantum dots can be used to monitor changes in wound PH to determine early wound status for timely treatment [41]. Phenylboric acid (PB)-conjugated C dots can enter macrophages in vitro with low toxicity, and the internalized C dots can be conjugated with antigens and deliver drugs or vaccines for cellular immunotherapy [42]. Therefore, it is important to evaluate the toxicity of CQDs in organisms for their safe application. Our study demonstrates that CQDs have good biocompatibility within a short exposure time; however, there were toxic effects under longer exposure times and higher concentrations. When the concentration reached 200 µg/mL, the osmotic pressure of the extracellular environment was significantly reduced, causing cell edema and deformation; by changing the surface charge of cells and adhering to the cell membrane to induce lipid peroxidation, the permeability of the membrane increased dramatically, increasing the intake of CQDs by *E. coli*. The CQDs will accumulate in the bacteria, leading to a more serious oxidative stress reaction and DNA damage in the cells. Finally, the cell walls of the bacteria will be damaged and the contents of the cells will be released, leading to cell death. Our study has comprehensively evaluated the toxicity and underlying mechanism of CQDs in simple organisms, with the aim of guiding future applications in the medical field.

## Data Availability

The data presented in this study are available in the article.
